# KNOWLEDGE OF AGING, NEGATIVE AGE BIAS, AND POSITIVE AGE BIAS: AGE GROUP DIFFERENCES

**DOI:** 10.1093/geroni/igz038.312

**Published:** 2019-11-08

**Authors:** Grace Caskie, Anastasia E Canell, Hannah M Bashian

**Affiliations:** Lehigh University, Bethlehem, Pennsylvania, United States

## Abstract

Attitudes towards aging include both positive and negative beliefs about older adults (Iverson et al., 2017; Palmore, 1999). Palmore’s (1998) Facts on Aging Quiz, a widely used assessment of knowledge about aging, also identifies common societal misconceptions about aging. Findings regarding age group differences in attitudes toward aging are mixed (Bodner et al., 2012; Cherry & Palmore, 2008; Rupp et al., 2005). The current study compared knowledge of aging, negative age bias, and positive age bias between young adults (18-35 years, n=268) and middle-aged adults (40-55 years; n=277). Middle-aged adults reported significantly greater average knowledge of aging than young adults (p=.019), although both groups had relatively low knowledge (MA: M=13.0, YA: M=12.2). Middle-aged adults also showed significantly less negative age bias (p<.001) and significantly more positive age bias than young adults (p=.026). Although the total sample was significantly more likely to be incorrect than correct on 23 of the 25 facts (p<.001), young adults were significantly more likely than middle-aged adults (p<.001) to respond incorrectly for only 2 of 25 facts. Both facts reflected greater negative age bias among young adults than middle-aged adults. These facts concerned older adults’ ability to work as effectively as young adults (fact 9) and frequency of depression in older adults (fact 13). Results demonstrate that age bias is not limited to young adults and may continue through midlife, though negative age bias in particular may be lower for individuals approaching older adulthood, which could have implications for their psychological and physical well-being.

